# Dispersive Magnetic Solid-Phase Extraction as a Novelty Sample Treatment for the Determination of the Main Aflatoxins in Paprika

**DOI:** 10.3390/toxins15020160

**Published:** 2023-02-15

**Authors:** María García-Nicolás, Natalia Arroyo-Manzanares, Pilar Viñas

**Affiliations:** Department of Analytical Chemistry, Faculty of Chemistry, Regional Campus of International Excellence “Campus Mare Nostrum”, University of Murcia, E-30100 Murcia, Spain

**Keywords:** magnetic solid-phase extraction, high-resolution mass spectrometry, mycotoxins, aflatoxins, polypyrrole nanocomposite, paprika

## Abstract

Dispersive magnetic solid-phase extraction (DMSPE) technique is proposed as a new sensitive and effective sample treatment method for the determination of aflatoxins in paprika samples. DMSPE was followed by ultrahigh-performance liquid chromatography and high-resolution mass spectrometry detection (UHPLC-HRMS) using a non-targeted acquisition mode for the detection of main aflatoxins (aflatoxin G1, G2, B1 and B2) and derivatives. DMSPE was based on the use of magnetic nanocomposite coated with polypyrrole (PPy) polymer and the main experimental parameters influencing the extraction efficiency in adsorption and desorption steps have been studied and optimized. Analyses were performed using 250 µL magnetic PPy nanocomposite into the sample solution, adsorbing the analytes in 30 min and desorbing them with ethyl acetate (2 mL) in 15 min. The method has been validated, obtaining quantification limits between 3.5 and 4.7 µg kg^−1^ and recoveries between 89.5–97.7%. The high recovery rate, wide detection range and the use for the first time of the reusable Fe_3_O_4_@PPy nanomaterial in suspension for solid food matrices, guarantee the usefulness of the method developed for adequate control of aflatoxins levels in paprika. The proposed methodology was applied for the analysis of 31 samples (conventional and organic) revealing the absence of aflatoxins in the samples.

## 1. Introduction

Dispersive magnetic solid-phase extraction (DMSPE) is a newly developed solid-phase extraction (SPE) technique in which the use of magnetic nanoparticles (MNPs) as adsorbents substitutes conventional sorbents [1,2]. MNPs improve the adsorption capacity of the analytes because of the high surface area, ensuring a fast mass transfer and allowing a desirable adsorbent separation from the solution by applying an external magnetic field, thanks to the superparamagnetism property [3], being their surface modification facilitated. External magnetic field application and separation allow the reversible agglomeration and redispersion of the magnetic adsorbent, thereby conveniently enabling, without additional centrifugation or filtration steps, the phase separation [4]. Therefore, the DMSPE technique is quick, since it reduces the required number of stages concerned, owing to simultaneous enrichment and separation of the compounds of interest occur at the same time, can be easily accomplished, and, most importantly, it is well-adapted to deal with large sample volumes, exhibiting great benefits in analyte isolation, preconcentration and enrichment [4]. Additionally, this approach addresses difficulties such as phase separation and column packing, both of which can be accomplished by external magnetic field application, and reduces the volume of toxic organic solvents and hazardous wastes produced, thus complying with Green Analytical Chemistry principles.

Aflatoxins (AFs) are recognized as the most significant carcinogenic and toxic mycotoxins that affect food derived from several species of *Aspergillus* (*Aspergillus nomius*, *Aspergillus parasiticus* and *Aspergillus flavus*). There are four main naturally occurring aflatoxins: AFB1 and AFB2 derived from *A. flavus* and AFG2 and AFG1 caused by both *A. parasiticus* and *A. flavus*. Toxicity levels associated with aflatoxin vary according to the types found, ranging in order of toxicity, carcinogenicity, and mutagenicity from AFB1 > AFG1 > AFB2 > AFG2 [5]. AFs are found in food since temperature, moisture, soil and storage conditions affect fungal growth before and after the harvest [6]. Therefore, given their frequent occurrence, the high toxicity and the low concentration of AFs, the potential for the development of fast, accurate, sensitive and trustable methods for AFs determination and screening in food matrices is of great importance.

Recently, the DMSPE technique has been applied using MNPs coated with different materials as sample treatment for the preconcentration and separation of aflatoxins in different food matrices. MNPs coated with polydopamine (PDA-MNPs) [7], 3-(trimethoxysilyl)-1-propanthiol (TMSPT) modified with 2-amino-5-mercapto-1,3,4-thiadiazole (AMT-TMSPT-MNPs) [8], PEGylated multi-walled carbon nanotubes (PEG-MWCNTs-MNP) [9], TMSPT modified with ethylene glycol bis-mercaptoacetate EGBMA-TMSPT-MNPs [10] and a new magnetic hollow bimetallic zinc/cobalt-based zeolitic imidazolate framework (HB-Zn/Co-ZIF-8) [11] have been used as sorbents in DMSPE for the assessment of AFG1, AFB1, AFM1, AFG2, AFB2, AFG2 and/or AFM2 in food liquid matrices including water, wine, milk and fruit juice. On the other hand, for AFB1, AFB2, AFG1 and AFG2 determination in oily matrices, edible and vegetable oils, nanoparticles such as magnetic graphene nanocomposite (Fe_3_O_4_/rGO) [12], PDA-coated MWCNTs [13] and Fe_3_O_4_@PDA MNPs have been used. However, this procedure has not only been studied for liquid or oily food samples, other agri-food matrices including maize, wheat, pistachio or fruit samples have been tested using different magnetic nanomaterials [11,14,15,16,17,18]. In addition, two types of nanomaterials synthesized using ionic liquids for B1, B2, G1 and G2 AFs determination in milk and pistachio samples [19,20] and an imprinted molecular polymer [21] in milk, rice and wheat flour samples have been used for the determination of the four major aflatoxins together with AFM1 and AFM2 with the DMSPE procedure. All these studies were carried out via a targeted approach, clearly pointing out the lack of other studies also doing non-targeted analysis in order to assess the presence of other aflatoxin-derived metabolites.

However, to date, this sample treatment has not been used on complex matrices as spices for the assessment of AFs. Among spice samples, paprika (*Capsicum annuum* L.) has emerged as one of the spices most widely employed in industrial food production and has gained considerable interest owing to its antioxidant attributes and also due to its high carotenoid content which makes it an excellent source of the color [22]. The European Commission of Agriculture and Rural Development [23] has recognized paprika produced in La Vera and Murcia (Spain) as a Protected Designation of Origin (PDO), thus being hugely important for local economies. 

Paprika mycotoxin contamination has been studied, with OTA and AFB1 showing the highest occurrence [24,25]. Recently, the presence of emerging mycotoxins has also been reported in paprika samples, particularly in enniatin B1 [26]. Current legislation of the European Union [27] sets maximum regulated levels of 5 µg kg^−1^ AFB1 and 10 µg kg^−1^ for the sum of AFB1, B2, G1 and G2 and otherwise the Food and Agriculture Organization (FAO) set the same regulation. 

Therefore, the present study represents a substantial technological innovation with respect to the current state of the art concerning the determination of mycotoxins and their metabolites in paprika samples, as it aims to carry out the development and evaluation of hybridization of sample preparations using a magnetic nanocomposite with new metabolic analytical techniques based on UHPLC-HRMS. The untargeted acquisition of major AFs in HRMS techniques combined with metabolomics strategies would provide a new evaluation of the presence of AFs in paprika samples, as well as the evaluation of the metabolization of these toxins. In addition, given the scarcity of studies doing non-targeted analysis of other metabolites derived from aflatoxins in paprika, the screening of these non-expected derivatives of this fungal toxic secondary metabolite class is carried out.

## 2. Results and Discussion

### 2.1. Characterization of Fe_3_O_4_@PPy Nanocomposite

Fe_3_O_4_@PPy synthesized nanocomposite was characterized using a field emission scanning electron microscope (FESEM) equipped with energy-dispersive X-ray spectroscopy (EDS). Elemental composition, nature and surface morphology were examined to ensure the correct synthesis of the developed nanomaterial.

To carry out these measurements, the Fe_3_O_4_@PPy nanoparticles were previously dehydrated for 10 min at 60 °C after assembly on special aluminum supports for SEM. After this time, they were coated with 5 nm of platinum in a vacuum sputter coater for characterization.

**Figure 1:**
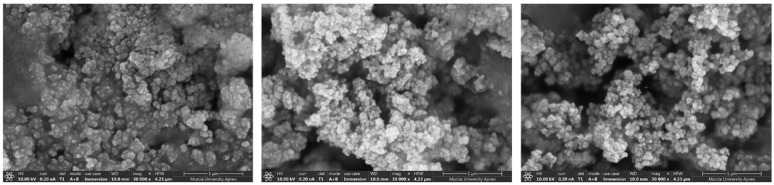
Triplicate FESEM scans of Fe_3_O_4_@PPy nanocomposite.

Three FESEM scans were carried out in three different areas of the nanocomposite surface to assess the homogeneity of the synthesized material. Figure 1 illustrates the FESEM surface images acquired. The morphological representation of Fe_3_O_4_@PPy nanocomposite at 1 µm scale reveals that Fe_3_O_4_ are being coated with PPy giving spherical grains shapes, with homogeneous particle size and distribution. 

To examine the elemental composition by EDS analysis, a voltage of 20 kV was used and atomic and weight percentages were calculated in triplicate. EDS measurements revealed peaks corresponding to N, O, Fe and C atoms. The standard deviation of the weight and atomic percentages of the four elucidated atoms obtained in each EDS measurement, was below 5 and 3%, respectively, which demonstrates the correct synthesis of the nanocomposite.

### 2.2. DMSPE Procedure Optimization

For optimization of the DMSPE procedure, the adsorption and desorption steps were studied in detail. In particular, for magnetic extractant phase preparation, different materials were tested and several parameters were evaluated: pH of the extraction medium, sample mass, amount of nanocomposite, volume and nature of desorption solvent. Preliminary experiments were performed in triplicate using 0.2 g of paprika sample spiked at 100 µg kg^−1^ with the four AFs (AFB1, AFB2, AFG1 and AFG2) and 5 mL of water. A 30 mg amount of each type of nanoparticle was added, followed by an adsorption time of 30 min and the desorption of the analytes in 1.5 mL of acetonitrile (MeCN) for 8 min under orbital shaking.

Firstly, the type of extraction phase was optimized, as it is crucial in the DMSPE sample preparation process. Ten different magnetic nanomaterials previously synthesized were tested based on ferrite (Fe_3_O_4_) coated with different materials: polystyrene (PS), silver (Ag), cellulose, chitosan, polypyrrole (PPy), multiwalled carbon nanotubes (MWCNTs), MWCNTs/PPy, polydopamine (PDA), 3-aminopropyl-triethoxysilane (APTS) and oleic acid. 

**Figure 2:**
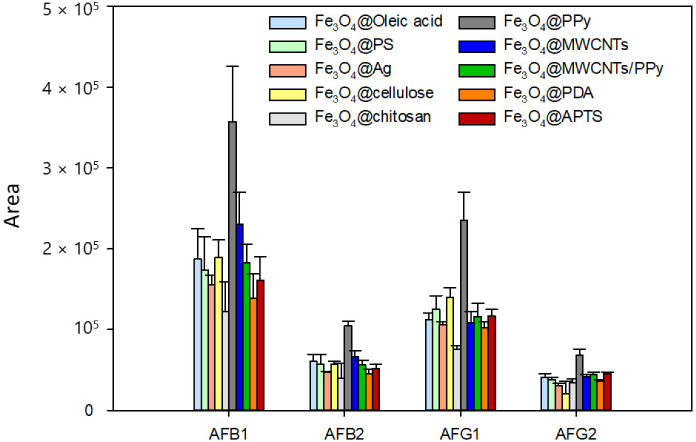
Influence of the nanocomposite type on the sensitivity of the aflatoxins.

Fe_3_O_4_@PS was evaluated because, in phenyl columns with silica-packing for HPLC, polystyrene is the principal bonding group and is known for its superior benefits in the sorting of multiple compounds as its high ratio of π-conjugated structures is very liable to be proper adsorbents for DMSPE [28]. Fe_3_O_4_@Ag [29] was tested due to silver is a highly active metal that has shown considerable thermal and mechanical strength and Fe_3_O_4_@cellulose [30] because cellulose presented a high biodegradability capability. Fe_3_O_4_@MWCNTs [31] was taken into account because of their high efficiency, porosity and wide surface area. Given the potential of PDA and chitosan as adsorbents, their good environmental stability, abundance and biocompatibility [32,33], Fe_3_O_4_@PDA and Fe_3_O_4_@chitosan nanoparticles were also tested. MNPs functionalized with PPy and APTS [34] were used as adsorbents due to their ease of synthesis, regeneration, environmental and mechanical stability and low cost. Finally, coating with oleic acid was tested oleic (Fe_3_O_4_@oleic acid) as oleic acid’s strongest advantage is the chemical bond between the amorphous iron oxide nanoparticles and the carboxylic acid group [35]. As can be seen in Figure 2, signals noticeably increase for all aflatoxins when using Fe_3_O_4_@PPy nanocomposite, while on the contrary lower enrichment is observed when the other nanomaterials were tested. This can be related to higher adsorption of the aflatoxins on the PPy polymer surface. Concretely, PPy on the surface of ferrite can overcome the decrease in active sites as a result of the presence of hydrogen bonding, π–π and hydrophobic interactions between PPy and target analytes [36]. Accordingly, the hydrophobic and π interaction between the aromatic rings of the AFs and the aromatic pyrrole rings of the Fe_3_O_4_@PPy is responsible for the efficient adsorption AFs on the nanocomposite surface. Then, PPy coating was compared in Fe_3_O_4_ and cobalt ferrite (CoFe_2_O_4_) core because of its excellent chemical and physical stability and its enhanced coercivity has been previously described [37]. However, the best results were obtained when the core was made of Fe_3_O_4_. 

Besides, similar nanomaterials with polypyrrole in their composition have previously reported positive results for the analysis of other organic analytes such as pyrethroids, benzoylurea insecticides and sulfonamides in food and environmental samples [36,38,39].

The influence of adding the magnetic material either as an aqueous suspension or as solid material of Fe_3_O_4_@PPy nanocomposite on the sensitivity of the method was examined. This comparison was carried out using 102 µL of an aqueous suspension and 100 mg as solid material of Fe_3_O_4_@PPy nanocomposite. It was observed that preconcentration was significantly higher when the extractant phase in the suspension form was added. Such behavior can be attributed to a reduced aggregation of the MNPs in suspension in comparison to when they are added in solid form. Consequently, the suspension form of the adsorbent phase was used, its volume optimized in the 100–400 µL range using the suspension form of 976 mg mL^−1^ MNPs concentration. Supplementary Figure S1 illustrates how sensitivity increased when a volume of 250 µL of nanocomposite suspension was used and that when the volume was higher than 250 µL, the sensitivity decreased. Therefore, 250 µL was chosen for further experiments. Supplementary Figure S2 shows the comparison of results when using the nanomaterial in a solid state after testing different masses of Fe_3_O_4_@PPy (30, 50, 100, 250 and 400 mg). The best results were obtained when 250 mg of nanoparticles are used for AFB1 and AFB2 and there were no significant differences between 250 mg and 400 mg for AFG1 and AFG2. So, the optimum amount of nanomaterial if used in the solid state would be 250 mg. This amount is approximately equivalent to the experiences made with the nanocomposite in suspension as 250 mg is 256 µL. Therefore, it can reconfirm that the best results are obtained when the Fe_3_O_4_@PPy is used in suspension, since comparing the optimum quantities obtained both in suspension and in the solid state again (in suspension, the areas obtained are higher).

Subsequently, the effect of ionic strength on the extraction efficiency in DMSPE was investigated by adding a percentage of sodium chloride. For this reason, 0 to 10% *m/v* values were tested. As demonstrated in Supplementary Figure S3, signals increased considerably over the studied range for all compounds, except for AFB1, for which in the presence of 5% or 10% *m/v* NaCl, the results obtained showed no significant differences. Thus, the addition of 10% of NaCl was selected for the DMSPE procedure. 

Then, pH influence of the extraction medium on sensitivity was assessed. The following pH levels were investigated: pH 3, 7 and 9. The acidic medium was adjusted using acetate/acetic acid buffer solution (0.1 M), the basic medium using phosphate buffer solution (0.1 M) and no adjustment in the sample preparation was necessary to reach pH 7. As expected, it was observed that there were significant differences in the pH values tested. At pH 3 and 9, the analytical signals were lower compared to those obtained at pH 7 (Supplementary Figure S4). Consequently, no pH adjustment of the extraction step was conducted because different paprika sample suspensions in 5 mL of water resulted in a pH of approximately 7.

Mycotoxin desorption from the nanocomposite was assessed using four organic solvents (methanol (MeOH), MeCN, ethyl acetate (EA) and chloroform) and also whether the presence of acid (5% formic acid) when MeOH and MeCN were used was beneficial. In this case, EA provided a significantly higher desorption efficiency, as can be seen in Supplementary Figure S5, being selected as the best solvent. EA is the least polar solvent of all solvents tested and provides the best desorption as its intermediate polarity is capable of disrupting the hydrophilic interactions between the hydrophilic structures of aflatoxins (terminal furan ring, the phenyl and the carbonyl moiety) and the PPy.

The volume of EA used for the desorption step was varied in the 1.5–3 mL range. As shown in Supplementary Figure S6, signals increased when 2 mL was used, except for AFB1, for which there were no significant differences using 2.5 or 3 mL for the desorption. Accordingly, 2 mL of EA was selected for the desorption of AFs.

**Figure 3:**
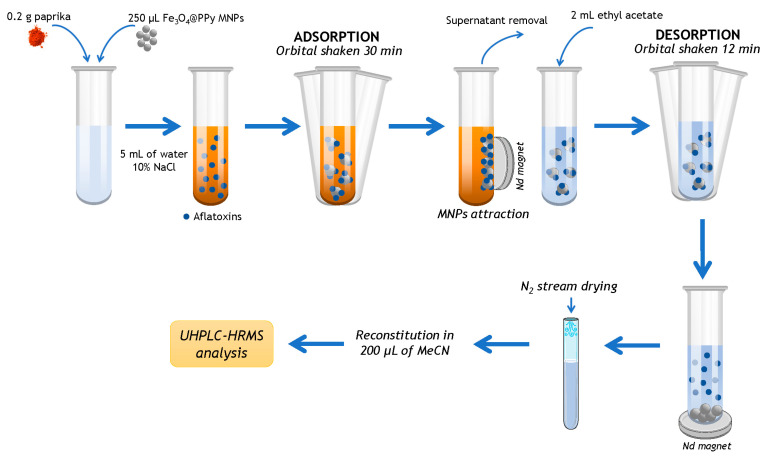
Diagram of optimal DMSPE-UHPLC-HRMS procedure used.

Finally, the desorption time required to desorb the analytes from the nanocomposite into the EA, under orbital shaking, was varied ranging from 1 to 16 min. As shown in Supplementary Figure S7, sensitivity was maximum when AFB1, AFG1 and AFG2 were 12 min in contact with the desorption solvent, while a modest increase in sensitivity was appreciated at 8 min for AFB2. Therefore, 12 min of desorption time was ultimately selected. Figure 3 shows the optimized DMSPE procedure.

### 2.3. Validation of the Analytical Method

The proposed method was evaluated in terms of suitability for the determination of AFB1, AFB2, AFG1 and AFG2 in paprika. For this reason, an evaluation of linear dynamic ranges, precision, trueness and limits of detection (LOD) and quantification (LOQ) was performed.

For the validation of the method, a sample of paprika free of aflatoxins was used, which was previously analyzed. Smoked paprika and normal paprika were compared for validation and no significant differences were found for either matrix effect or analyte extraction, so smoked paprika was further selected. This sample was fortified at different concentration levels of AFs, homogenized and left to stand for 1 h to allow interaction between the mycotoxins and paprika matrix. Afterwards, the proposed analytical procedure was applied.

For quantification, a matrix-matched calibration was conducted by spiking at six concentration levels (between 3.5 and 50 μg kg^−1^) of the aflatoxins, a sample of paprika free of the analytes. Duplicate preparation and injections were carried out for each concentration level, considering the peak area as an analytical signal. Calibration results are shown in Table 1. Supplementary Figure S8 shows an LC-HRMS chromatogram of studied aflatoxins in spiked paprika at 10 µg kg^−1^. The R^2^ values showed good linearity for the range studied. With regard to LODs and LOQs, LODs varied between 1.0 and 1.4 µg kg^−1^, corresponding to AFG1 and AFG2, respectively. By comparison, LOQs were in the 3.5–4.7 µg kg^−1^ range, also corresponding to AFG1 and AFG2, respectively (Table 1). As laid down in the current legislation for aflatoxin contamination in spices [27], the LOQs obtained are very satisfactory and would allow us to quantify aflatoxin contents below the limits established in the legislation. Specifically, the LOQ obtained for AFB1 is 3.7 µg kg^−1^, being the maximum content allowed in the legislation for this mycotoxin of 5 µg kg^−1^. For the sum of AFB1, B2, G1 and G2 a maximum level of 10 µg kg^−1^ is allowed.

Both repeatability and reproducibility of the method were obtained by calculating the relative standard deviation (RSD) of peak areas and the results are shown in Table 1. The RSD values ranged between 5.3–7.6% and 5.3–7.8% for repeatability and reproducibility, respectively, in agreement with current legislation for aflatoxins contamination in spices [27].

Trueness in terms of recovery results was expressed as mean values of nine experiments, which are shown in Table 2. Recoveries were in the range of 81.9–99.4% with RSD values from 0.7–10.5% complying with the current legislation requirements for aflatoxins in spices [27]. The matrix effect was assessed in terms of signal suppression/enhancement (SSE). Signal suppression was detected, obtaining SSE values ranging from 82.0–87.6% (Table 2) and confirming the need to use matrix-matched calibration. 

### 2.4. Comparison with Other Methods

The newly developed method was further compared with other reported methods for the determination of AFs on other food samples to evaluate the potential of the analytical platform proposed using Fe_3_O_4_@PPy nanocomposite as an adsorbent for DMSPE and the results are summarized in Supplementary Table S1.

It can be observed that the proposed method showed a comparable RSD range and recovery rate to the previously reported methods. Moreover, this study has the advantage of a wide detection range and is the first one that uses the Fe_3_O_4_@PPy nanomaterial in suspension for solid food matrices. 

In addition, to compare the developed method more thoroughly, a short validation was performed using the same chromatographic conditions on an HPLC-MS/MS. So, LODs, LOQs, linear dynamic range and matrix effect parameters were calculated as described in the validation section. In this case, LODs varied between 0.7 and 0.9 µg kg^−1^, corresponding to AFG1 and AFB2, respectively. By comparison, LOQs were in the 2.2–3.0 µg kg^−1^ range, also corresponding to AFG1 and AFG2, respectively (Supplementary Table S2). Compared to the proposed method, these LODs and LOQs are similar to those obtained using the UHPLC-HRMS equipment with the same sample treatment. Conversely, higher signal suppression was detected, obtaining SSE values ranging from 60.1–63.2%. Hence, the developed DMSPE-UHPLC-HRMS method is a viable method for the determination of main AFs contamination in paprika samples.

### 2.5. Commercial Paprika Samples Analysis

Method applicability was lastly assessed by means of analyzing different samples of commercial paprika produced in “La Vera”, “Murcia” and “Espelette”. Specifically, a total of 31 samples were analyzed in triplicate, including 27 conventional and 4 organic samples, of which 12 were expired. A targeted analysis of the data acquired was used to investigate AFG1, AFG2, AFB1 and AFB2 presence in paprika samples. However, none of the four most important aflatoxins was detected in any of the samples. 

Subsequently, with the aim of presenting a comprehensive understanding of aflatoxins presence in paprika samples, the occurrence of other important aflatoxins, for which reference standards were not available, was investigated in the same paprika samples data files (Supplementary Table S3) for which reference standards were not available. Concretely, 15 AFs were researched (Aflatoxin B2a, Aflatoxin G2a, Aflatoxin GM1, Aflatoxin M1, Aflatoxin M2a, Aflatoxin M2, Aflatoxin M4, Aflatoxin P1, Aflatoxin P2, Aflatoxin Q2a, Aflatoxicol H1, Aflatoxin Q1, Aflatoxicol, Aflatoxicol B and Aflatoxicol M1), resulting in none detection in any of samples studied. All these aflatoxins have been previously described in other food and agri-food matrices [40,41].

Finally, other metabolites related to the aflatoxin biosynthesis pathway or described as degradation products of AFB1 and AFB2 were also screened in the samples (Supplementary Table S3). Specifically, sterigmatocystin (ST), O-methyl-sterigmatocystin (OMST), dihydrosterigmatocystin (DHST) and dihydro-O-methylsterigmatocystin (DHOMST) were the metabolites sought belonging to the aflatoxin metabolic pathway [42]. On the other hand, 10 aflatoxin degradation products, previously described using low resolution [43,44], were investigated for the first time using high resolution. However, no contamination by these metabolites was found in the samples.

## 3. Conclusions

This study presented a novel method for the simultaneous analysis of AFs in paprika samples. In this work, Fe_3_O_4_@PPy nanocomposite was successfully synthesized and used as an extraction phase adsorbent for DMSPE. It is expected that the magnetic PPy adsorbent fabricated by a combination of hydrogen-bonding, hydrophobic and π–π interactions, efficiently adsorb AFs. Fe_3_O_4_@PPy combines properties such as being environmentally and mechanically stable, easy to synthesize, regenerate and cheap, and being able to be reused five times according to the developed study, all of which makes it a really convenient preconcentration method. Combined with UHPLC-HRMS, the developed fast, simple operation, reliable and sensitive sample preparation technique shows low LODs and LOQs, high recovery rates, good accuracy and precision for the determination of the main AFs quantitatively and the screening of other derivatives of this fungal toxic secondary metabolite class. 

## 4. Materials and Methods 

### 4.1. Reagents and Standards

Individual mycotoxin standards were acquired from Sigma-Aldrich (St. Louis, MO, USA). AFG1, AFB1, AFG2 and AFB2 were prepared as separated stock solutions at 1 mg L^−1^ in acetonitrile (MeCN) and placed in storage at −20 °C. Methanol (MeOH), ethanol, ethyl acetate (EA) and MeCN of chromatographic grade were supplied by ChemLab (Zedelgem, Belgium). 

For the synthesis of PPy nanocomposite, iron (III) chloride hexahydrate (FeCl_3_·6H_2_O), ammonia solution, iron (II) chloride tetrahydrate (FeCl_2_·4H_2_O), sodium hydroxide, pyrrole and sodium perchlorate reagents were all acquired from Sigma-Aldrich. A Milli-Q system from Millipore (Bedford, MA, USA) was used to obtain the ultra-pure water. 

Ammonium acetate and formic acid were used for mobile phase composition. In addition, during the DMSPE procedure optimization, sodium chloride and chloroform were used. All the reagents above mentioned were supplied by Sigma-Aldrich.

Before chromatographic analysis, sample filtration was carried out using 0.22 µm × 25 mm nylon syringe filters purchased from Agela Technologies (New York, NY, USA). 

### 4.2. Instrumentation and Software

UHPLC-HRMS analyses were performed using an Agilent 1290 Infinity II Series HPLC (Agilent Technologies, Santa Clara, CA, USA) with a high-speed binary pump (thereby comprising the UHPLC system) coupled to an Agilent 6550 QTOF Mass Spectrometer using an Agilent jet stream dual electrospray (AJS-Dual ESI) source. MassHunter workstation software from Agilent Technologies (Version B.08.00, Santa Clara, CA, USA) was used for data acquisition and MS-DIAL (Version 4.80, RIKEN, Yokohama, Japan) was used for data interpretation. 

For sample processing, an Xcelvap air-drying system from Horizon Technology (Salem, MA, USA) and an orbital shaker IKA-KS-130-Basic (Staufen, Germany) were used. The permanent magnets employed were blocks consisted of Nd-Fe-B with a strength, dimensions and weight of 33 kg, 50 × 15 × 15 mm and 86 g, respectively. Such magnets were purchased from Supermagnete (Gottmadingen, Germany).

Image data were acquired by field emission scanning electron microscopy (FESEM) with ApreoS Thermo FESEM (ThermoFisher Scientific, MA, USA) and energy-dispersive X-ray spectroscopy (EDS) analyses were conducted using EDAX-Ametek (EDAX, AMETEK Materials Analysis Division, Mahwah, NJ, USA).

For data treatment, Sigmaplot 13.1 (Systat, Software Inc., San Jose, CA, USA) was used as statistic software. 

### 4.3. Fe_3_O_4_@PPy Nanocomposite Synthesis

The synthesis of the Fe_3_O_4_@PPy magnetic nanocomposite was carried out according to the procedure described by Asgharinezhad et al. [31] with some modifications. Once the ferrite magnetic core (Fe_3_O_4_) was synthesized, Fe_3_O_4_@PPy nanocomposite synthesis was carried out. With this objective, 0.5 g of the dried Fe_3_O_4_ was dissolved in 200 mL of deionized water under continuous agitation at pH 9 for 5 min. A volume of 0.25 mL pyrrole was added and the mixture was shaken for 10 min. Afterwards, an addition of 0.5 g of sodium perchlorate to the mixture was carried out and then it was subjected to stirring for 5 min; and 25 mL of 18 mg mL^−1^ FeCl_3_·6H_2_O solution was added to the mixture dropwise while stirring. The preparation was left overnight under orbital shaking at room temperature to allow the polymerization reaction. Then, the nanocomposite obtained was washed with deionized water and ethanol several times until neutral pH. Finally, the nanocomposite suspension was prepared in 20 mL water, which is equivalent to a 976 mg mL^−1^ nanocomposite concentration. On the other hand, the nanocomposite as solid material is obtained by drying overnight at 70 °C.

### 4.4. Samples

Different paprika samples produced in “La Vera”, “Murcia” and “Espelette” were purchased from local markets. Specifically, 31 samples of which 4 were organic and 27 were conventional samples were analyzed. Of the conventional samples, 12 were expired. Moreover, within the set of samples, the varieties of hot paprika, sweet paprika and smoked paprika were found. 

The purchased bulk samples were transferred into sterile plastic containers, covered with aluminum foil to prevent degradation by light and kept in storage until analysis, the remaining samples were left in their sealed opaque commercial containers. All samples were stored at room temperature.

### 4.5. Sample Treatment

For sample preparation, an amount of 0.2 g of paprika, 5 mL of ultra-pure water containing 10% NaCl and the nanocomposite in suspension were placed into a test tube. Concretely, a volume of 250 µL (244 mg) of Fe_3_O_4_@PPy nanocomposite suspension was added and the resulting mixture was submitted to orbital stirring for 30 min at room temperature. The nanocomposite was then magnetically attracted with a neodymium magnet, applied externally, and the supernatant solution was discarded. Aflatoxins were desorbed by adding 2 mL of ethyl acetate to the enriched magnetic material and the mixture was further shaken orbitally for 12 min at ambient temperature. Afterwards, the separation of the nanocomposite from the supernatant was again performed using the magnet. Then, the supernatant solution collected was evaporated until dryness using an N_2_ stream (1200 mbar) at 35 °C, reconstituted in 200 µL of MeCN and vortexed for 1 min. The reconstituted extract was filtered with a 0.2 µm nylon filter before injection in the UHPLC-QTOF-MS system. For recovery experiments, the same sample treatment was performed, previously fortifying paprika samples at two concentration levels (10 and 25 μg kg^−1^) using an appropriate volume of mixed aflatoxin solution. Spiked samples were left to stand for 1 h to allow interaction between the mycotoxins and paprika matrix and solvent evaporation. 

### 4.6. UHPLC-HRMS Analysis

The chromatographic separation of main AFs was achieved using a ZORBAX RRHD Eclipse Plus C18 column, 2.1 × 100 mm i.d., 1.8 μm particle size using a 0.3 μm Agilent Technologies inline filter. 

Water: MeOH (95:5, *v*/*v*) (solvent A) and MeOH: water (95:5, *v*/*v*) (solvent B) both containing 0.3% formic acid and 5 mM ammonium acetate, were used as mobile phases. A flow rate of 0.4 mL min^−1^ was used. The gradient profile was set as follows: 0–0.5 min: 40% B; 0.5–5.5 min: 40–70% B; 5.5–6 min: 70–40% B; 6–8 min: 40% B. Twenty microliters were injected into the system. The autosampler and column were at a temperature of 5 °C and 35 °C, respectively. 

Positive mode operation of the mass spectrometer was used. The drying gas flow was set to 16 L min^−1^ at a temperature of 130 °C and the nebulizer gas pressure was established at 30 psi. On the other hand, the sheath gas temperature was 300 °C and the flow rate was set at 11 L min^−1^. The following voltages were used for the capillary spray, fragmentor, nozzle and 1 RF Vpp octopole: 4000, 360, 500 and 750 V. 

MS scans in the range of 50–1500 *m*/*z* were set for data acquisition. Moreover, an MS scan collection was configured for an extended dynamic 2 GHz range mode with 2675 transients/spectrum, 3 spectra/s and 333.3 ms/spectrum. All ions mode was used for non-targeted data acquisition. The following collision energies were used in each cycle: 0, 10 and 40 V.

Data from UHPLC-HRMS were transformed into analysis Base Framework (ABF) formats and further processed using MS-DIAL which comprises peak selection, deconvolution, compound identification and peak alignment. A targeted data analysis was then performed for quantitation of the main AFs according to retention time, exact mass MS and MS/MS data. Supplementary Table S4 summarizes the UHPLC-HRMS setups to monitor AFB1, AFB2, AFG1 and AFG2 ions as well as their precursor ions (*m*/*z*), retention times, and the instrumental error associated with the measurements in ppm. The difference between the experimental and theoretical *m*/*z* divided by the theoretical *m*/*z* value and multiplied by 10^6^, was used to calculate the instrumental error.

### 4.7. HPLC-MS/MS Analysis

Secondary short validation was carried out in a 1200 high-performance liquid chromatograph (HPLC), where AFs chromatographic separation was performed using an Agilent InfinityLab Poroshell 120 EC-C18 column, 4.6 × 150 mm i.d. and 2.7 µm of particle size. Mobile phases used were A (H_2_O with 0.1% of HCOOH and 2 mM of HCOONH_4_) and B (MeOH containing 0.1% of HCOOH). The flow rate was 0.5 mL min^−1^. The gradient profile was set as follows: 30% B, increased linearly to 99% B after 20 min, which was held until 35 min, then decreased progressively to reach again 30% B at 37 min and was held for 8 min. Twenty microliters were injected into the system. 

Analyses were conducted setting positive electrospray ionization (ESI) mode in a 6410 triple quadrupole mass spectrometer detector (QqQ-MS/MS) from Agilent Technologies coupled to the HPLC system. The MS data were obtained using the mode of multiple reaction monitoring (MRM) and the source parameters were set as follows: 350 °C gas temperature, 3000 V capillary voltage, 40 psi nebulizer pressure and 9 L min^−1^ gas flow. Fragmentor voltages and collision energies (EC) were tested from 120 to 180 V and from 4 to 100 V, respectively. 

### 4.8. Method Validation

The developed method was validated for AFB1, AFB2, AFG1 and AFG2 mycotoxins in paprika. The validation was performed according to Commission Regulation (EC) No 401/2006 [45].

Linearity was calculated by least-square regression. The LODs and LOQs were calculated for a signal-to-noise ratio (S/N) of 3 and 10, respectively. The precision of the proposed method was evaluated in terms of repeatability and intermediate precision (intraday and interday precision, respectively). The whole procedure was applied to three spiking samples at two concentration levels (10 and 25 μg kg^−1^) on the same day and injected in triplicate to assess repeatability. In addition, intermediate precision was estimated by analyzing three samples, which were also spiked at the same two concentration levels, on four different days. 

In order to test the trueness of the proposed method, recovery studies were carried out in paprika samples free of mycotoxins. Samples were fortified at two concentration levels (10 and 25 μg kg^−1^) and the optimized method was applied. The injection was carried out in triplicate. Recoveries were calculated as 100 * (concentration found/real concentration added).

Finally, the SSE effect due to matrix interferences for each aflatoxin, and the slopes of linear calibration built in a blank matrix and a neat solvent were compared. Therefore, this effect was quantified as follows: SSE (%) = 100 * (Slope spiked cleaned-up extract/slope spiked matrix-free injection solvent). The entity of matrix effect is calculated as 100 − SSE (%) where values above 100 imply signal enhancement and values below 100 indicate signal suppression.

## Figures and Tables

**Table 1:** Validation data for the determination of aflatoxins in paprika.

Mycotoxin	Equation	Linearity (μg kg^−1^)	Linearity R^2^	LOD (μg kg^−1^)	LOQ (μg kg^−1^)
AFB1	y = 5440x − 10,919	3.7–50	0.995	1.1	3.7
AFB2	y = 3636x − 11,161	3.9–50	0.993	1.2	3.9
AFG1	y = 5686x − 16,997	3.5–50	0.990	1.0	3.5
AFG2	y = 1982x − 15,273	4.7–50	0.998	1.4	4.7
	**Repeatability, %RSD (*n* = 9)**		**Intermediate precision, %RSD (*n* = 12)**		
	**10 μg kg^−1^**	**25 μg kg^−1^**	**10 μg kg^−1^**		**25 μg kg^−1^**
AFB1	5.8	5.5	7.0		7.1
AFB2	7.6	7.1	7.2		7.7
AFG1	6.4	6.7	7.5		7.8
AFG2	5.3	5.9	5.6		5.3

**Table 2:** Recoveries with RSD given in parentheses, and matrix effect (%) of the AFs in paprika.

	Recoveries (%) (*n* = 9)		Matrix Effect (%)
Mycotoxin	10 μg kg^−1^	25 μg kg^−1^	
AFB1	99.4 (0.7)	81.9 (7.6)	87.6
AFB2	95.9 (4.8)	98.7 (1.4)	84.7
AFG1	95.3 (4.6)	96.9 (3.0)	84.4
AFG2	88.5 (10.0)	88.8 (10.0)	82.0

## Data Availability

The data presented in this study are available in this article and supplementary materials.

## Supplementary Materials

The following supporting information can be downloaded at: https://www.mdpi.com/article/10.3390/toxins15020160/s1, Figure S1: Influence of the Fe_3_O_4_@PPy suspension volume on the sensitivity of the analytes; Figure S2: Influence of the Fe_3_O_4_@PPy mass on the sensitivity of the analytes; Figure S3: Influence of the NaCl content in the extraction medium on AFs preconcentration efficiency; Figure S4: Influence of the pH on AFs preconcentration efficiency; Figure S5: Influence of the desorption solvent nature on AFs preconcentration efficiency; Figure S6: Influence of the desorption solvent volume on AFs preconcentration efficiency; Figure S7: Influence of the desorption time on AFs preconcentration efficiency; Figure S8. LC-HRMS chromatogram of AFB1, AFB2, AFG1 and AFG2 in spiked paprika at 10 µg kg^−1^; Table S1: Comparison of the novel proposed method with other methods for the determination of AFs in food samples; Table S2: Validation data for the determination of aflatoxins in paprika using HPLC-MS/MS; Table S3: AFs and their derivatives investigated using untargeted processing; Table S4: UHPLC-HRMS parameters of the target aflatoxins.
