# Supplementary material for: Dispersive Magnetic Solid-Phase Extraction as a Novelty Sample Treatment for the Determination of the Main Aflatoxins in Paprika
Source: Toxins (Basel). 2023 Feb 15;15(2):160. doi: 10.3390/toxins15020160 (PMC9959555; doi:10.3390/toxins15020160)
Supplement: Supplementary file 1 [file toxins-15-00160-s001.zip › toxins-2204879-supplementary.pdf]

Dispersive Magnetic Solid-Phase Extraction as a Novelty Sample Treatment for the Determination of the Main Aflatoxins in Paprika

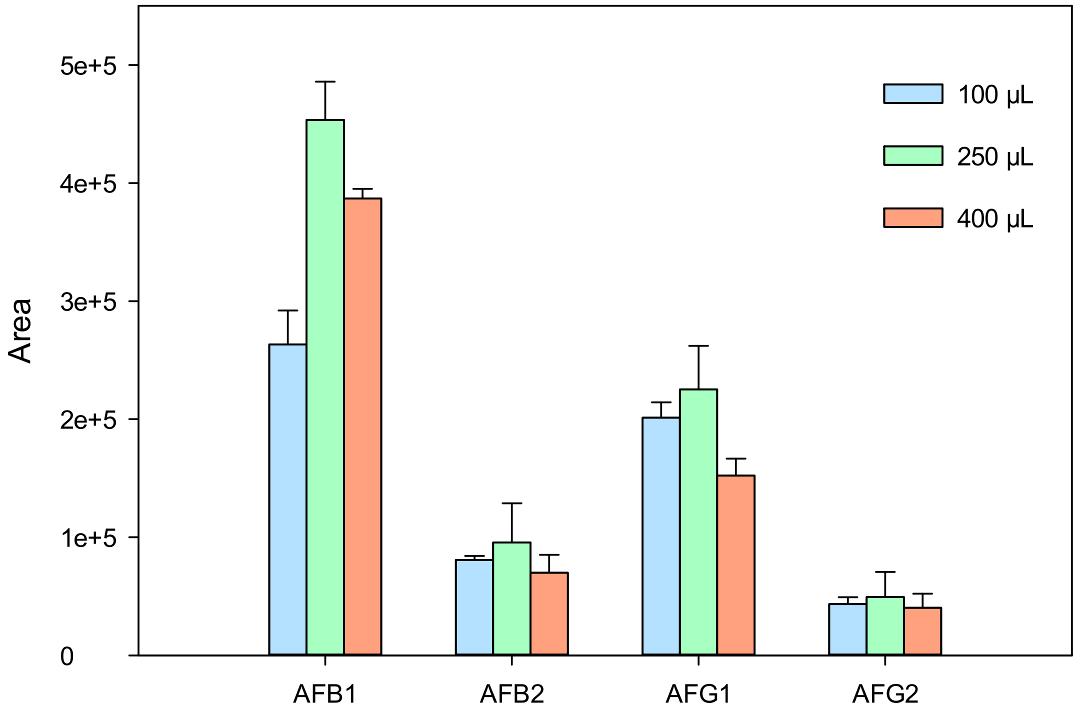

Figure S1. Influence of the Fe_3O_4@PPy suspension volume on the sensitivity of the analytes.

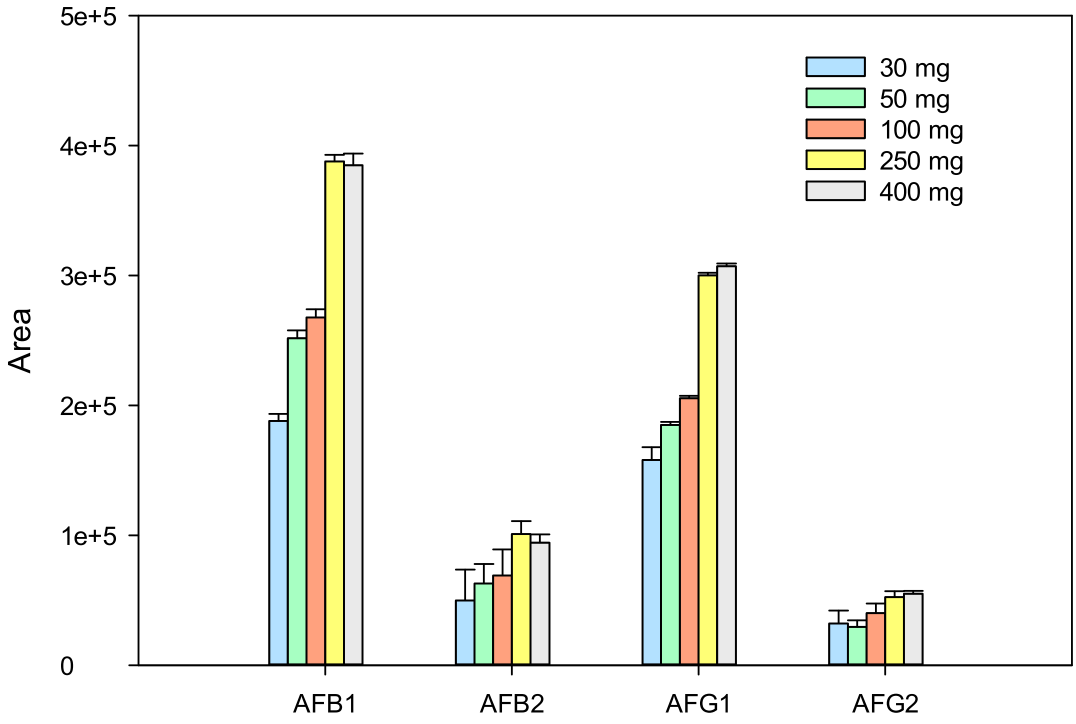

Figure S2. Influence of the Fe_3O_4@PPy mass on the sensitivity of the analytes.

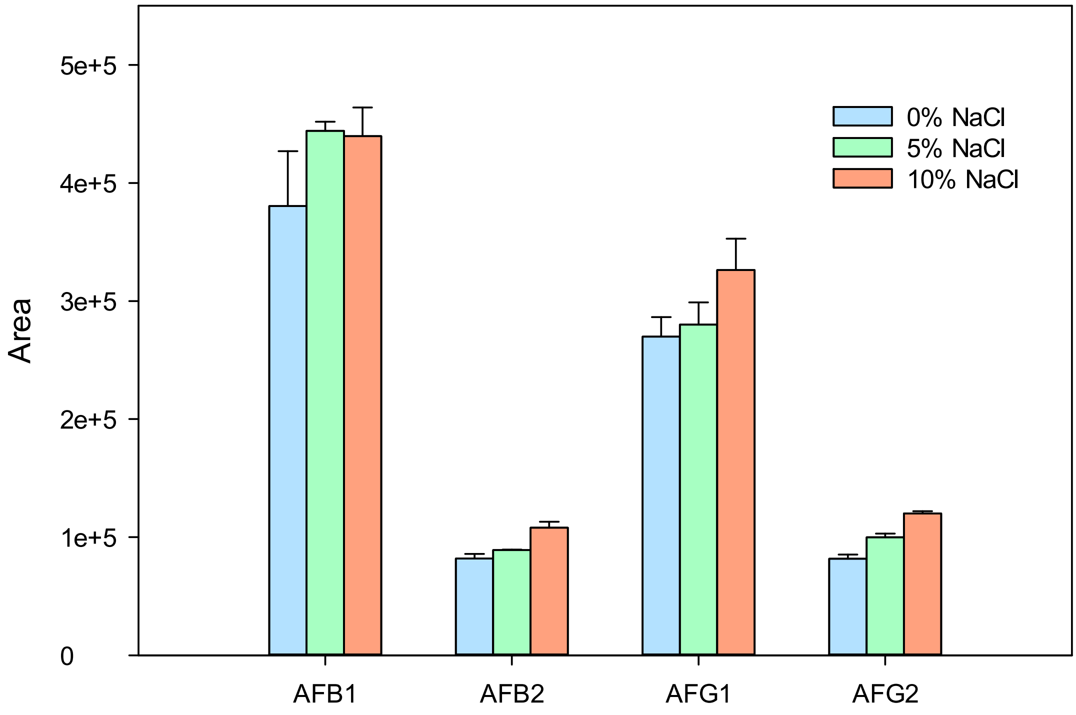

Figure S3. Influence of the NaCl content in the extraction medium on AFs preconcentration efficiency.

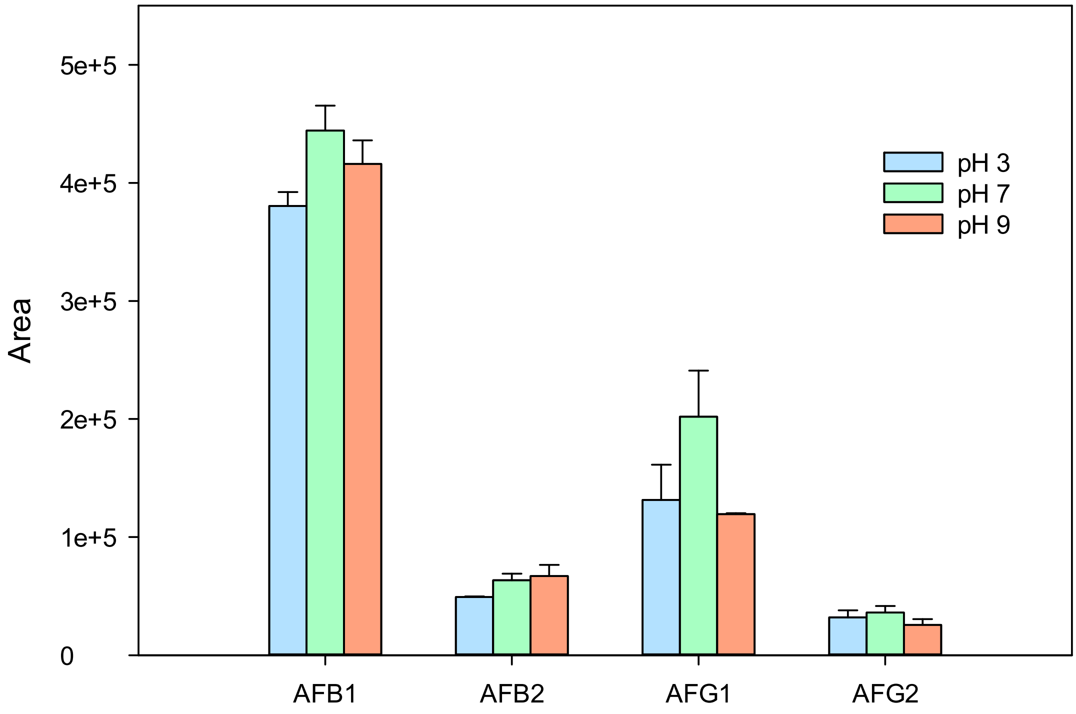

Figure S4. Influence of the pH on AFs preconcentration efficiency.

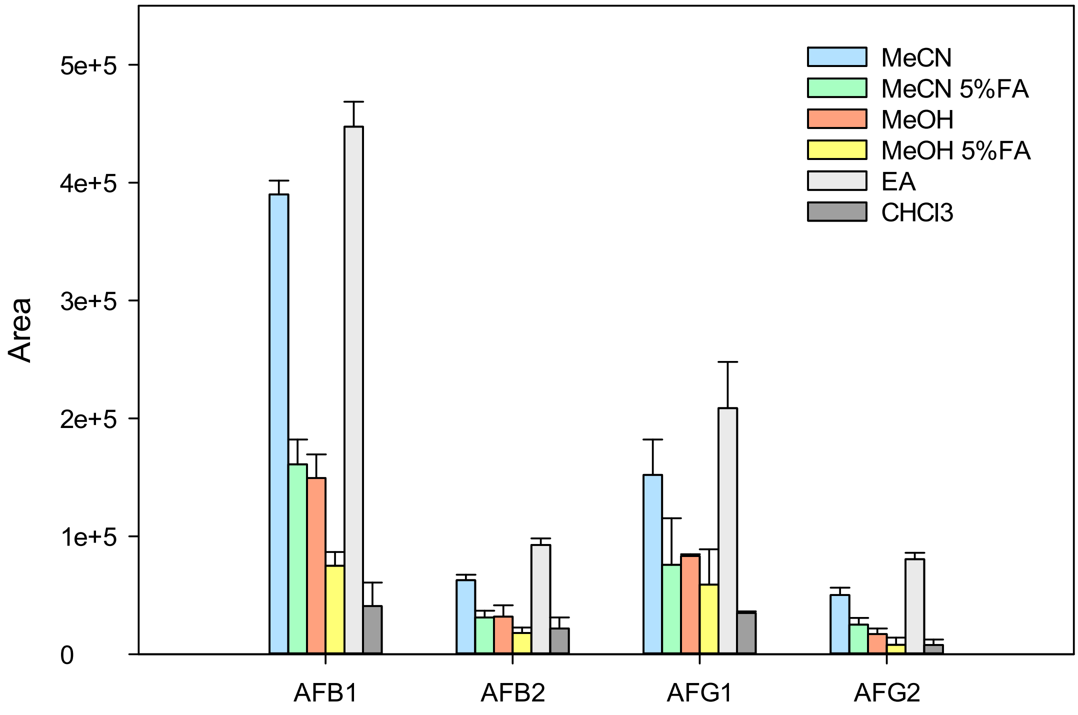

Figure S5. Influence of the desorption solvent nature on AFs preconcentration efficiency.

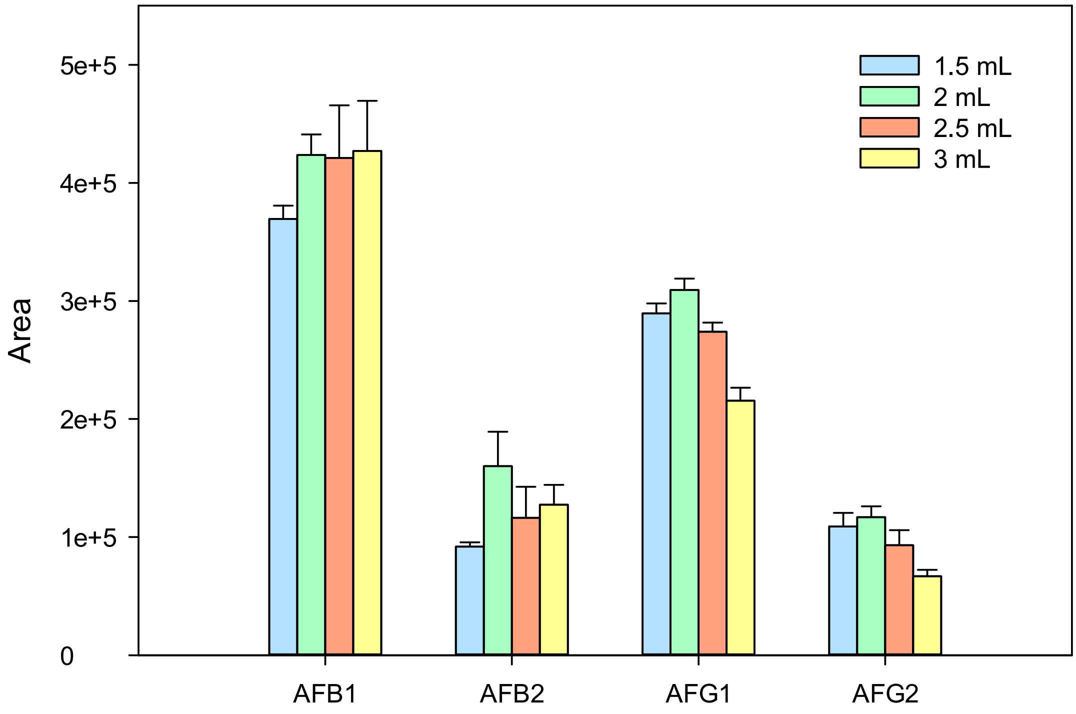

Figure S6. Influence of the desorption solvent volume on AFs preconcentration efficiency.

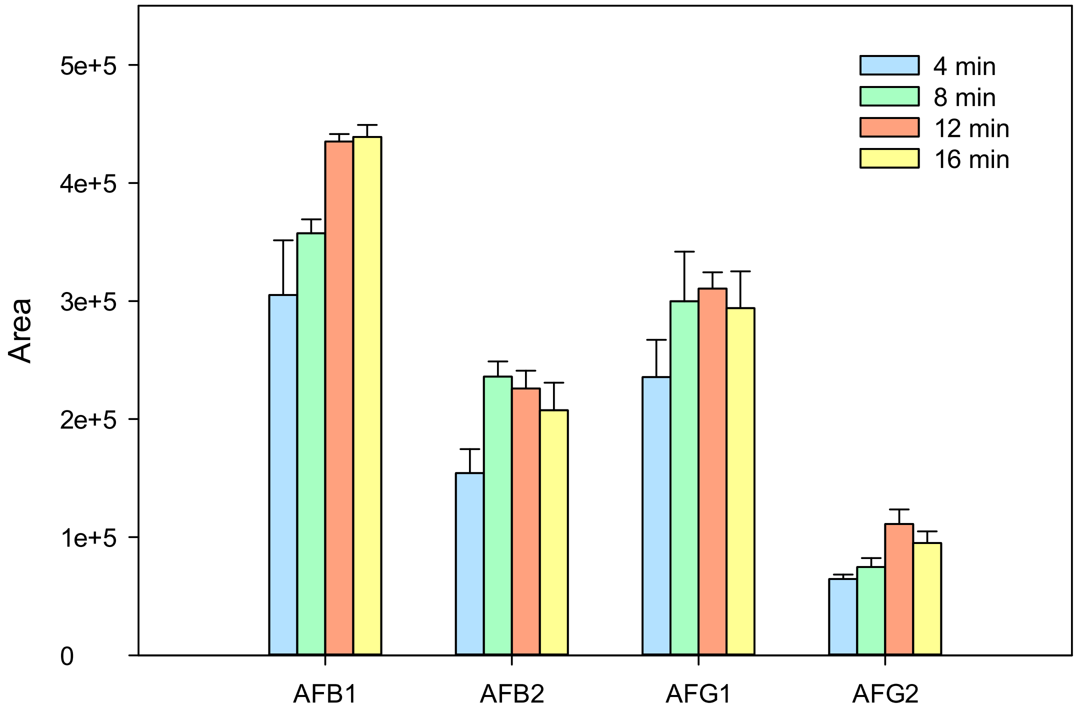

Figure S7. Influence of the desorption time on AFs preconcentration efficiency.

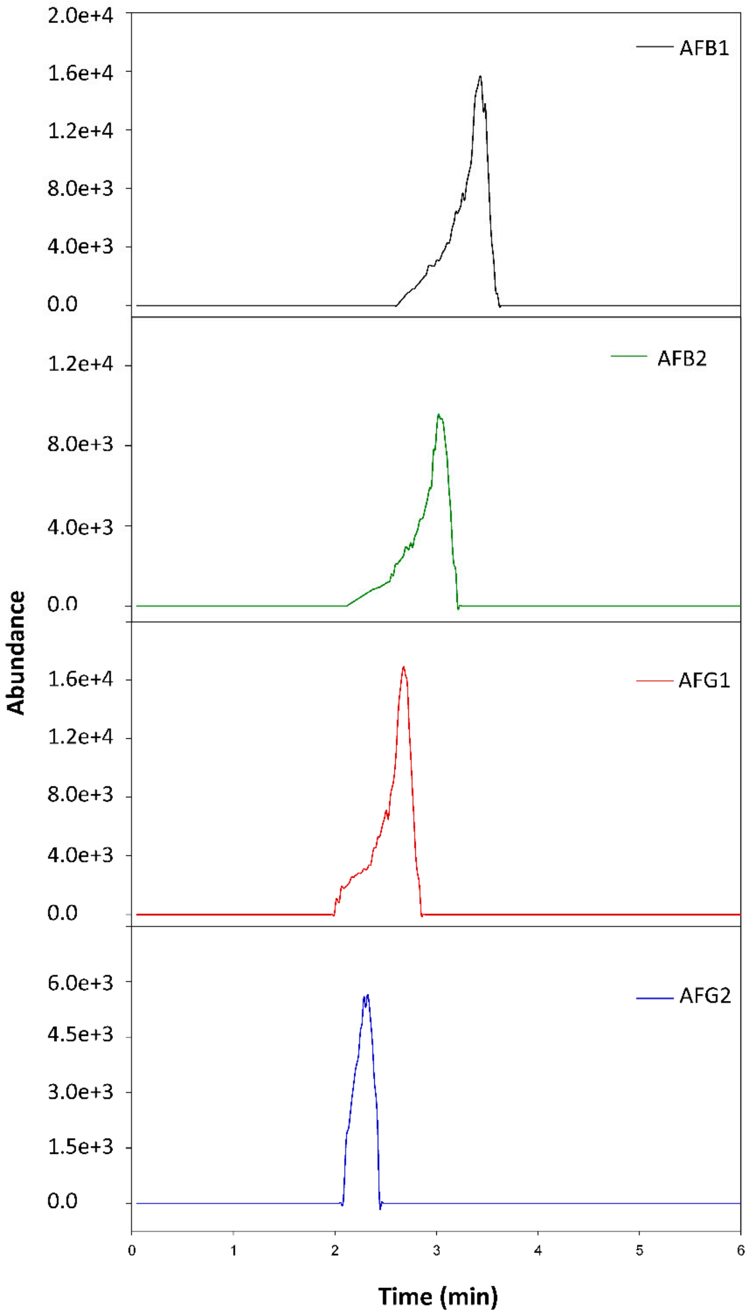

Figure S8. LC-HRMS chromatogram of AFB1, AFB2, AFG1 and AFG2 in spiked paprika at 10 μg kg^{-1}.

Table S1. Comparison of the novel proposed method with other methods for the determination of AFs in food samples.

AFs	Matrix	NPs type	Determination technique	Sorbent amount	Method time	Organic solvent consumed	Linearity	Recovery (%)	RSD (%)	LOD	Ref
B1 B2 G1 G2	Fruit	HB-Zn/Co-ZIF-8	UHPLC-IT-MS	40 mg	60 min	150 μL of 0.1%FA–ACN	5-100 ng mL^{-1} (B1, G1, G2)<br>1-50 ng mL^{-1} (B2)	75.1-102.4	5.3-13.6	0.18-1.50 ng mL^{-1}	(Wang et al., 2020)
B1 B2 G1 G2	Pistachio	Sp-M-Dp	HPLC-FLD	150 mg	20 min	1 mL ACN	2-10 ng g^{-1}	72.0-95.6	5.6–7.3	0.02-0.07 ng g^{-1}	(Karami-Osboo, Ahmadpoor, Nasrollahzadeh, & Maham, 2022)
B1 B2	Maize	MAMs	HPLC-PCD-FLD	NI	7 min	5 mL of BB and 1 mL of eluting buffer/MeOH (20:80, v/v)	0.5-50 μg kg^{-1}	83.6-97.8	1.6-8.00	10-25 pg mL^{-1}	(Liu et al., 2018)
B1 B2 G1 G2	Wheat	EGBMA-TEOS-MNPs	IAC-HPLC-FLD	100 mg	12 min	2 mL of a mixture of Me_2CO/MeCN/CH_2Cl_2 (1:1:2, v/v) and 2 mL of 0.5 mM Triton X-100 in 15% (v/v) ACN/water.	0.1-50 ng mL^{-1}	92.0-105.0	0.8-2.6	0.03 ng mL^{-1}	(Hanif, Allahyari, Pourghazi, & Amoli-diva, 2015)
B1	Wheat	Fe_3O_4-MWCNTs-NH_2	HPLC-DAD	6 mg	40 min	0.2 mL of Me_2CO	1-100 ng g^{-1}	88.8-96.0	2.1–2.8	0.15 ng g^{-1}	(Li, Zhang, & Shi, 2018)
B1 B2 G1 G2	Pistachio	AMT–TMSPT-MNPs	HPLC -FLD	150 mg	8 min	2 mL Me_2CO/MeCN/CH_2Cl_2 (1:1:2, v/v/v)	0.10-15 ng mL^{-1} (B1, G1)<br>0.04-3 ng mL^{-1} (B2, G2)	92.5-103.2	2.3-5.9	0.014-0.037 μg kg^{-1}	(Hashemi & Taherimaslak, 2014)
M2 M1 G2 G1 B2 B1	Rice and wheat flour	MIP-capped MGO/MOF-808	HPLC-FlD	5 mg	25 min	0.5 mL ACN, 300 μL phosphate buffer (0.2 M, pH 7) and 250 μL GQDs.	0.03-5 ng mL^{-1}	97.3-98.6	0.9-2.9	0.009-3.69 ng mL^{-1}	(Alilou, Amirzehni, & Eslami, 2021)

B1 B2 G1 G2	Pistachio	MIL-101(Cr) MIL-101(Cr)/Fe_3O_4@SiO_2@PTU	HPLC-FLD	60 mg	6 min	215 µL CH_2Cl_2/ACN/ Me_2CO (2:1:1, v/v)	0.2-4 ng g^{-1} (B1, G1)<br>0.1-3.5 ng g^{-1} B2<br>0.3-04 ng g^{-1} G2	93.4-97.8	6.4-12.8	0.02–0.09 ng g^{-1}	(Bagher, Hamid, & Ghasempour, 2020)
B1 B2 G1 G2	Paprika	Fe_3O_4@PPy	UHPLC-HRMS	244 mg	42 min	2 mL of EA	3.7 – 50 µg kg^{-1} (B1)<br>3.9 – 50 µg kg^{-1} (B2)<br>3.5 – 50 µg kg^{-1} (G1)<br>4.7 – 50 µg kg^{-1} (G2)	81.9-99.4	5.3-7.8	1.0 - 1.4 µg kg^{-1}	This work

Table S2. **V**alidation data for the determination of aflatoxins in paprika using HPLC-MS/MS.

Mycotoxin	Equation	Linearity (µg kg^{-1})	Linearity R^2	LOD (µg kg^{-1})	LOQ (µg kg^{-1})	Matrix effect (%)
AFB1	y = 2624 x + 1521	2.3 - 50	0.992	0.7	2.3	61.9
AFB2	y = 639.5 x + 173	3.0 - 50	0.997	0.9	3.0	61.1
AFG1	y = 2232 x + 302	2.2 - 50	0.988	0.7	2.2	63.2
AFG2	y = 373.6 x + 108	2.5 - 50	0.998	0.8	2.5	60.1

Table S3. AFs and their derivatives investigated using untargeted processing.

Aflatoxin	Molecular Formula	*m/z*
Aflatoxin B2a	$C_{17}H_{14}O_7$	330.0739
Aflatoxin G2a	$C_{17}H_{14}O_8$	346.0689
Aflatoxin GM1	$C_{17}H_{12}O_8$	344.0532
Aflatoxin M1	$C_{17}H_{12}O_7$	328.0583
Aflatoxin M2	$C_{17}H_{14}O_7$	330.0739
Aflatoxin M2a	$C_{17}H_{14}O_8$	346.0689
Aflatoxin M4	$C_{17}H_{12}O_7$	328.0583
Aflatoxin P1	$C_{16}H_{10}O_6$	298.0477
Aflatoxin P2	$C_{16}H_{12}O_6$	300.0634
Aflatoxin Q1	$C_{17}H_{12}O_7$	328.0583
Aflatoxin Q2a	$C_{17}H_{14}O_8$	346.0689
Aflatoxicol	$C_{17}H_{14}O_6$	314.0790
Aflatoxicol B	$C_{17}H_{14}O_6$	314.0790
Aflatoxicol H1	$C_{17}H_{14}O_7$	330.0739
Aflatoxicol M1	$C_{17}H_{14}O_7$	330.0739
Biosynthesis pathway metabolites		
Sterigmatocystin	$C_{18}H_{12}O_6$	325.0707
O-methyl-sterigmatocystin	$C_{19}H_{14}O_6$	339.0863
Dihydrosterigmatocystin	$C_{18}H_{14}O_6$	327.0863
Dihydro-O-methylsterigmatocystin	$C_{19}H_{16}O_6$	341.1020
Degradation products		
DP1	$C_{16}H_{12}O_4$	269.0808
DP2	$C_{17}H_{10}O_6$	311.0550
DP3	$C_{17}H_{10}O_7$	327.0499
DP4	$C_{17}H_{16}O_6$	317.1020
DP5	$C_{16}H_{12}O_6$	301.0707
DP6	$C_{16}H_6O_5$	279.0288
DP7	$C_{16}H_{10}O_5$	283.0601
DP8	$C_{16}H_{22}O_5$	295.1540
DP9	$C_{16}H_{20}O_5$	293.1384
DP10	$C_{16}H_{20}O_6$	309.1333

DP11	$C_{16}H_{18}O_6$	307.1176
DP12	$C_{16}H_{16}O_7$	321.0929

Table S4. UHPLC-HRMS parameters of the target aflatoxins.

**Compound**	**RT[a] (min)**	**Formula**	**Precursor ion (*m*/*z*)**	**Error (ppm)**	**Product ion 1 (*m*/*z*)**	**Product ion 2 (*m*/*z*)**
AFG2	2.319	$C_{17}H_{14}O_7$	331.0812	1.3	245.1	313.1
AFG1	2.679	$C_{17}H_{12}O_7$	329.0656	-0.37	243.1	311.1
AFB2	3.021	$C_{17}H_{14}O_6$	315.0863	-0.59	286.9	259
AFB1	3.432	$C_{17}H_{12}O_6$	313.0707	-0.75	241	284.9

[a] RT: retention time
